# A Prognostic Nomogram of Colon Cancer With Liver Metastasis: A Study of the US SEER Database and a Chinese Cohort

**DOI:** 10.3389/fonc.2021.591009

**Published:** 2021-02-26

**Authors:** Chuan Liu, Chuan Hu, Jiale Huang, Kanghui Xiang, Zhi Li, Jinglei Qu, Ying Chen, Bowen Yang, Xiujuan Qu, Yunpeng Liu, Guangwei Zhang, Ti Wen

**Affiliations:** ^1^ Department of Medical Oncology, The First Hospital of China Medical University, Shenyang, China; ^2^ Key Laboratory of Anticancer Drugs and Biotherapy of Liaoning Province, The First Hospital of China Medical University, Shenyang, China; ^3^ Liaoning Province Clinical Research Center for Cancer, Shenyang, China; ^4^ Medical College, Qingdao University, Qingdao, China; ^5^ Smart Hospital Management Department, The First Hospital of China Medical University, Shenyang, China

**Keywords:** colon cancer with liver metastasis, overall survival, prognostic factors, nomogram, external validation

## Abstract

**Background:**

Among colon cancer patients, liver metastasis is a commonly deadly phenomenon, but there are few prognostic models for these patients.

**Methods:**

The clinicopathologic data of colon cancer with liver metastasis (CCLM) patients were downloaded from the Surveillance, Epidemiology and End Results (SEER) database. All patients were randomly divided into training and internal validation sets based on the ratio of 7:3. A prognostic nomogram was established with Cox analysis in the training set, which was validated by two independent validation sets.

**Results:**

A total of 5,700 CCLM patients were included. Age, race, tumor size, tumor site, histological type, grade, AJCC N status, carcinoembryonic antigen (CEA), lung metastasis, bone metastasis, surgery, and chemotherapy were independently associated with the overall survival (OS) of CCLM in the training set, which were used to establish a nomogram. The AUCs of 1-, 2- and 3-year were higher than or equal to 0.700 in the training, internal validation, and external validation sets, indicating the favorable effects of our nomogram. Besides, whether in overall or subgroup analysis, the risk score calculated by this nomogram can divide CCLM patients into high-, middle- and low-risk groups, which suggested that the nomogram can significantly determine patients with different prognosis and is suitable for different patients.

**Conclusion:**

Higher age, the race of black, larger tumor size, higher grade, histological type of mucinous adenocarcinoma and signet ring cell carcinoma, higher N stage, RCC, lung metastasis, bone metastasis, without surgery, without chemotherapy, and elevated CEA were independently associated with poor prognosis of CCLM patients. A nomogram incorporating the above variables could accurately predict the prognosis of CCLM.

## Introduction

Among all malignant tumors, the incidence and mortality of colon cancer (CC) ranked fourth and fifth worldwide in both genders, respectively (1–3). In recent years, owing to the development of multiple therapeutic strategies [operation, chemotherapy, neoadjuvant chemoradiotherapy, and radiotherapy (RT)], the prognosis of CC has been improved. For example, Hu et al. (4) found that the duration of adjuvant chemotherapy may be related to improved DFS of CC patients. Besides, the adjuvant RT also benefits the cause-specific survival of CC patients (5). On these bases, the 5-year survival rate of T1–T2 stage CC patients was up to 89.9%, while 71.3% in the T3–T4 stage (5). However, nearly 13% of CC patients have been found to have distant metastases at the time of diagnosis, of which the survival rate of five years is only 13.3% (6, 7). Among all distant metastases patterns, liver metastases are the most common, accounting for about one-third. In general, colorectal cancer (CRC) is usually studied as a whole cohort. However, CC patients are more likely to metastasize to the liver than rectal cancer (RC), which may be attributed to the different directions of blood metastasis of CC and RC and results in different metastasis patterns. Thus, patients of CC with liver metastasis (CCLM) is a unique subset that deserves further study. Of all CCLM. only 10–25% are eligible for surgery and more than half of them will develop recurrence within three years, so it is obvious that these patients have a worse prognosis than patients without liver metastasis (8–10). Therefore, it is necessary to explore the prognostic factors to accurately predict the prognosis of CCLM patients for individual planning.

In previous studies, some prognostic factors for CC patients were reported, including stage and metastatic status, which revealed the association between clinicopathologic features and the prognosis of CC patients (11–14). Nevertheless, there is no large cohort-based study in exploring prognostic factors of CCLM patients. Therefore, in the present study, we intended to identify overall survival (OS)-related variables of CCLM patients and establish a nomogram as a more intuitive tool. Besides, as for different advanced patients, the effect of treatments is significantly different, so we included treatments as prognostic factors to discover the benefit of treatment to patients and avoid over-medication.

Additionally, the prognosis of different CC pathology is different. For example, in patients with stage III CC, proximal colon cancer was found to be worse than the distal (15). Hence, we also conducted the subgroup analyses of the left-sided and right-sided colon cancers and other subgroups to validate the efficacy of our prognostic nomogram. Finally, we also included an external validation to further verify the nomogram, which would provide treatment advice for patients with different risks and help clinical decision-making.

## Methods

### Population Selection

The Surveillance, Epidemiology and End Results (SEER) is a cancer database based on the US population, which collected data on cancer patients from 18 registries and covered more than 30% of the population (16). The data of patients in the present research were downloaded from the SEER∗Stat 8.3.6 software. Patients with histological diagnosis as CCLM from 2010–2015 were included. According to the histology and site codes, patients with adenocarcinoma (8,140–8,147, 8,210–8,211, 8,220–8,221, 8,260–8,263), mucinous adenocarcinoma (MAC) (8,480–8,481), and signet ring cell carcinoma (SRCC) (8,490) and the tumor site of colon (site code: C18.0 and C18.2–18.9) were included. Meanwhile, patients were excluded if: (1) the information of race, histological grade, AJCC T stage, AJCC N stage, accurate tumor size, tumor site, surgery, radiotherapy, chemotherapy, carcinoembryonic antigen (CEA), and metastatic status of liver, lung, bone, and brain is unknown; (2) not the first tumor; (3) survival time < 1 month; (4) age at diagnosis < 18 years old. All included CCLM patients were randomly divided into a training set (70%) and an internal validation set (30%). The training set was used to determine the independent prognostic factors for CCLM patients and establish the prognostic nomogram, while the internal validation sets were used to validate the nomogram.

To further validate our nomogram responsibly, patients diagnosed as CCLM from August 1998 to May 2019 in The First Hospital of China Medical University were used to form the external validation set. This validation set included 101 CCLM patients who were recruited according to inclusion and exclusion criteria the same as the training cohort. The time of the last follow-up was June 2020. This study was approved by the institutional review board of The First Hospital of China Medical University.

### Variable Collection

The variables included in the present study were age at diagnosis, race, gender, tumor site, histological type, tumor size, histological grade, AJCC T status, AJCC N status, CEA, metastasis sites (lung, brain, and bone), and information of therapy (surgery, radiotherapy, and chemotherapy). The optimal cut-off values of age and tumor size were determined by the X-tile software (17), and the results showed that the best cut-off values of age were 61 and 76 years old, while the optimal cut-off values of the tumor size were 4.6 and 6.1 cm. In our research, the primary outcome was OS, which was defined as the time interval between the day of diagnosis and death for all causes.

### Statistical Analysis

The statistical analysis in our study was performed in SPSS 25.0 or R software (Version 3.6.1). A p value<0.05 (two-sided) was considered statistically significant. Firstly, the univariate Cox analysis was used to determine OS-related variables in the training set. Then, the variables with a p-value <0.05 in the univariate Cox analysis were included in the multivariate Cox analysis to identify the independent prognostic factors of CCLM patients. After that, a nomogram was established by the “rms” package in R software based on those independent prognostic factors. Meanwhile, the time-dependent receiver operating characteristic (ROC) curves at 1-, 2-, and 3-years were plotted, and the corresponding time-dependent area under the curve (AUC) values were used to evaluate the discrimination of the nomogram. Besides, the corresponding calibration curves were established to show the calibration of the nomogram, and decision curve analysis (DCA) was performed to show the clinical benefit of the nomogram. Furthermore, based on the risk score and X-tile software, the optimal cut-off values were determined and all patients were stratified into low-, middle-, and high-risk groups. The Kaplan-Meier survival curve was generated to show the difference in OS between the three groups. During the validation of the nomogram, the total points of each patient in two validation sets were calculated according to the nomogram developed in the training set, then Cox regression in this cohort was performed using the total points as a factor, and finally, the C-index, calibration curve and DCA were derived based on the regression analysis (18).

Furthermore, to confirm that the effectiveness of the nomogram was better than a single factor, the ROC curves of all independent prognostic factors were generated. Subgroup analysis was performed in left-side CC (LCC), right-side CC (RCC), liver-only metastasis, multiple metastases, CEA-elevated, CEA-normal, grade I–II, and grade III–IV. The Kaplan-Meier survival curves for each subgroup were generated.

## Results

### Clinicopathologic Characteristics

According to the criteria of inclusion and exclusion, a total of 5,700 CCLM patients were included, which were divided into a training set (n=3,992) and an internal validation set (n=1,708). The Chi-square test showed that there was no significant difference between the two sets (**Table 1**). The average age of these patients was 62.05 ± 13.18 (range: 21–108) years old, and 54.4% of patients were male. Besides, the CEA was elevated in most patients. In comparison, the pathological type in most CCLM patients is adenocarcinoma, accompanied by deep infiltration (T3–T4), grade II, and surgery-received, and the distribution of which was similar to that of CC patients (19). Notably, we found that most CCLM patients have a relatively higher proportion of lymph node metastasis (N1–N2) (80.8%) compared with CC patients (36.2%) (19).

**Table 1:** Baseline characteristics of CCLM patients in the training and internal validation groups.

Variables	Total	Training group (n = 3,992)	Validation group (n = 1,708)
Age(year)			
<61 61–76	2,594 2,241	1,821 1,558	773 683
>76	865	613	252
Gender			
Male	3,101	2,187	914
Female	2,599	1,805	794
Tumor size(cm)			
<4.6	2,237	1,558	679
4.6–6.1	1,731	1,213	518
>6.1	1,732	1,221	511
Race			
White	4,214	2,951	1,263
Black	975	672	303
Other	511	369	142
Site			
LCC	2,580	1,802	778
RCC	3,120	2,190	930
T stage			
T1–2	414	292	122
T3–4	5,286	3,700	1,586
N stage			
0	1,095	765	330
1	2,164	1,502	662
2	2,441	1,725	716
Grade			
I II	226 3,999	152 2,793	74 1,206
III IV	1,230 245	877 170	353 75
Type			
Adenocarcinoma	5,257	3,672	1,585
Mucinous adenocarcinoma Signet ring cell carcinoma	411 32	294 26	117 6
Lung metastasis			
Yes	906	649	257
No	4,794	3,343	1,451
Bone metastasis			
Yes	155	107	48
No	5,545	3,885	1,660
Brain metastasis			
Yes	30	19	11
No	5,670	3,973	1,697
Surgery			
Yes	5,242	3,655	1,587
No	458	337	121
Radiation			
Yes	189	127	62
No	5,511	3,865	1,646
Chemotherapy			
Yes	4,368	3,040	1,328
No	1,332	952	380
CEA			
Normal	985	684	301
Elevated	4,715	3,308	1,407

### Identification of Prognostic Factors of CCLM Patients in the Training Set

To identify OS-related variables, sixteen variables were included in the univariate Cox analysis. The result showed that age, tumor size, race, tumor site, histological type, grade, CEA, AJCC T status, AJCC N status, extrahepatic metastasis (lung, brain, and bone), and treatments (surgery and chemotherapy) were identified as OS-related variables (**Table 2**). Then, the multivariate Cox analysis was performed and the result indicated that higher age, the race of black, larger tumor size, higher grade, histological type of mucinous adenocarcinoma and signet ring cell carcinoma, higher AJCC N status, RCC, lung metastasis, bone metastasis, without surgery, without chemotherapy, and elevated CEA were independently associated with poor OS of CCLM patients (**Table 2**).

**Table 2:** Univariate and multivariate Cox regression analysis of CCLM patients based on clinicopathological characteristics in the training cohort.

	Univariate Cox				Multivariate Cox			
	HR	95% CI		P	HR	95%CI		P
Age								
<61				0.000				0.000
61–76	1.312	1.210–1.424		0.000	1.163	1.071–1.264		0.000
>76	2.460	2.220–2.726		0.000	1.776	1.590–1.984		0.000
Size								
<4.6cm				0.000				0.000
4.6-6.1cm	1.141	1.043–1.248		0.004	1.072	0.979–1.173		0.134
>6.1	1.353	1.239–1.477		0.000	1.266	1.159–1.384		0.000
Race								
Black				0.111				0.006
White	0.902	0.818–0.994		0.037	0.850	0.770–0.937		0.001
Other	0.908	0.783–1.054		0.206	0.863	0.742–1.003		0.055
Sex								
Female								
Male	0.976	0.906–1.050		0.513				
Site								
LCC								
RCC	1.545	1.434–1.665		0.000	1.339	1.238–1.447		0.000
Histological type								
Adenocarcinoma				0.000				0.001
Mucinous adenocarcinoma	1.322	1.157–1.510		0.000	1.202	1.050–1.377		0.008
Signet ring cell carcinoma	2.756	1.827	4.158	0.000	1.932	1.274–2.929		0.002
Grade								
I				0.000				0.000
II	1.057	0.869–1.286		0.577	1.167	0.959–1.420		0.123
III	1.641	1.338–2.013		0.000	1.653	1.346–2.030		0.000
IV	1.781	1.383–2.294		0.000	1.834	1.422–2.366		0.000
T								
T1–2								
T3–4	0.921	0.799–1.061		0.255				
N								
0				0.000				0.000
1	1.130	1.014–1.259		0.026	1.361	1.218–1.521		0.000
2	1.449	1.305–1.608		0.000	1.805	1.611–2.022		0.000
CEA								
Normal								
Elevated	1.538	1.385–1.707		0.000	1.612	1.450–1.791		0.000
Surgery	0.520	0.459–0.589		0.000	0.399	0.348–0.458		0.000
Radiation	0.978	0.792–1.208		0.835				
Chemotherapy	0.343	0.316–0.372		0.000	0.356	0.326–0.389		0.000
Bone	1.951	1.593–2.390		0.000	1.530	1.244–1.882		0.000
Brain	2.160	1.321–3.532		0.002				
Lung	1.606	1.460–1.765		0.000	1.404	1.273–1.549		0.000

### Development and Validation of the Prognostic Nomogram

**Figure 1:**
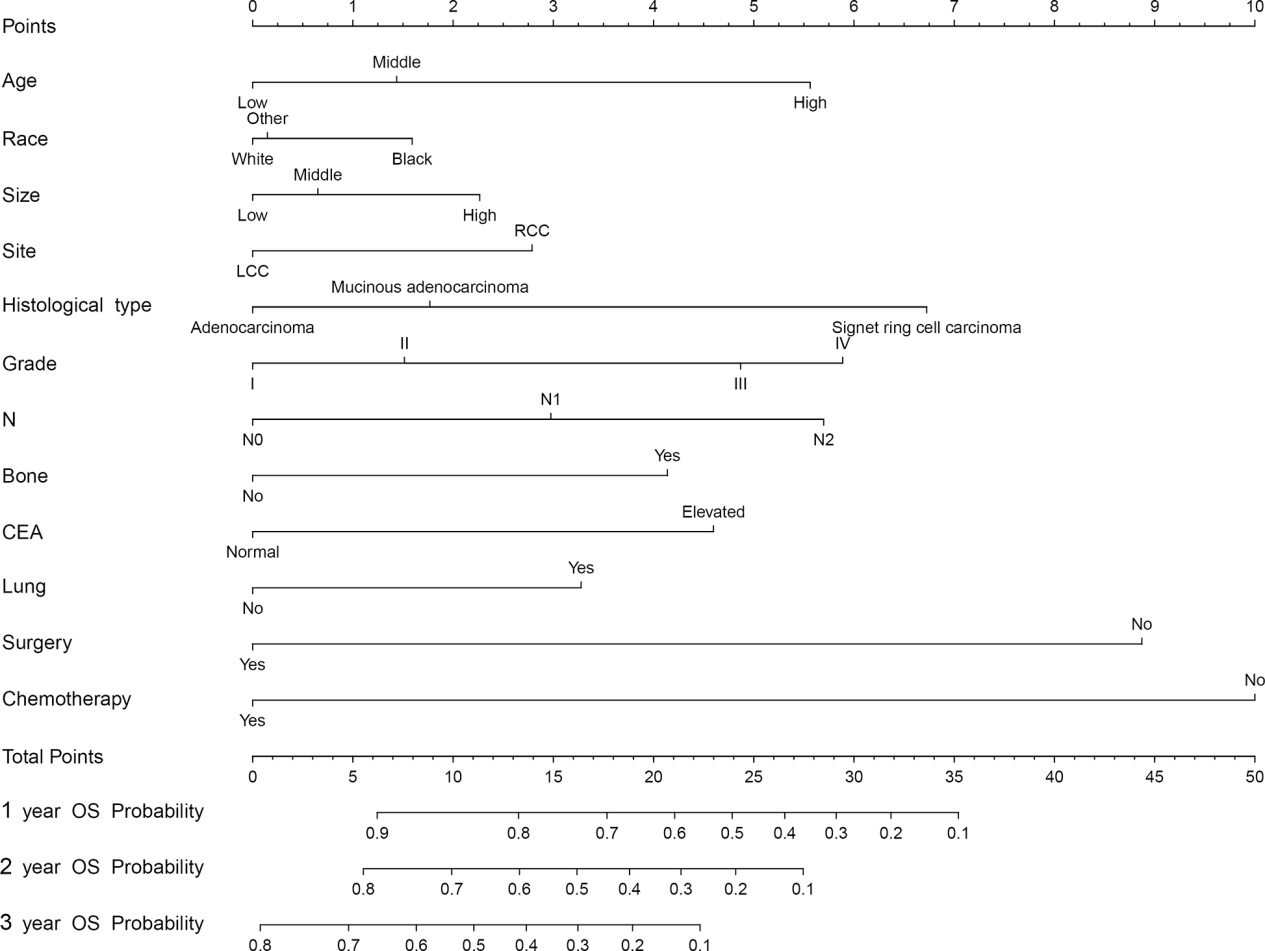
The nomogram for predicting the prognosis of CCLM patients.

To predict the OS of CCLM, a nomogram was developed based on all independent OS-related factors from the training set (**Figure 1**). Meanwhile, the time-dependent ROC curves showed that the AUC values in 1-, 2-, and 3-years were 0.792, 0.769, and 0.763, respectively, which suggested the favorable discrimination of the nomogram (**Supplementary Figure S1**). Then, the AUC values in 1-, 2- and 3-years were 0.754, 0.747, and 0.751 in the internal validation set and 0.725, 0.738 and 0.700 in the external validation set, respectively. Besides, the calibration curves indicated that the nomogram has a strong calibration. Furthermore, DCA was performed and the results indicated that the nomogram can serve as an effective tool for clinical practice (**Supplementary Figure S1**).

### Risk Stratification for CCLM Patients

**Figure 2:**
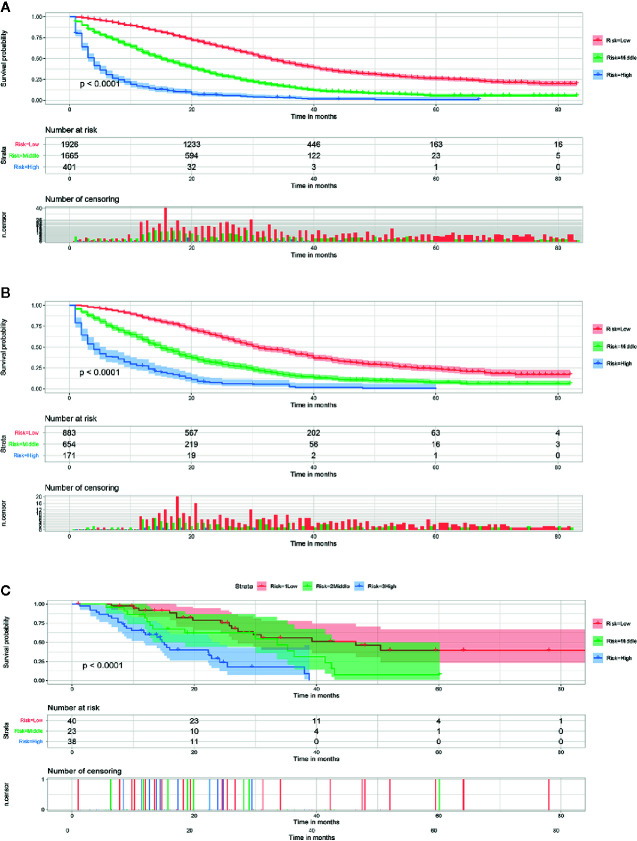
Survival curves showed the survival status classified by our nomogram of the training set **(A)**, internal validation set **(B)**, and external validation set **(C)** in CCLM patients.

Using our established prognostic nomogram, CCLM patients can be divided into high-, middle- and low-risk groups. As shown in **Figure 2A**, the results of Kaplan-Meier survival analysis with log-rank test suggested that there existed different survival patterns among patients in the three risk groups. Moreover, patients in both validation sets were also divided into three risk groups with the result of X-tile. We can see that patients of the low-risk group had a better prognosis than patients in the high-risk group (P<0.0001) (**Figures 2B, C**). The above results indicate that the nomogram can divide CCLM patients into three groups with different prognosis to provide a reference for treatment.

### Risk Stratification for Subgroup Analysis

**Figure 3:**
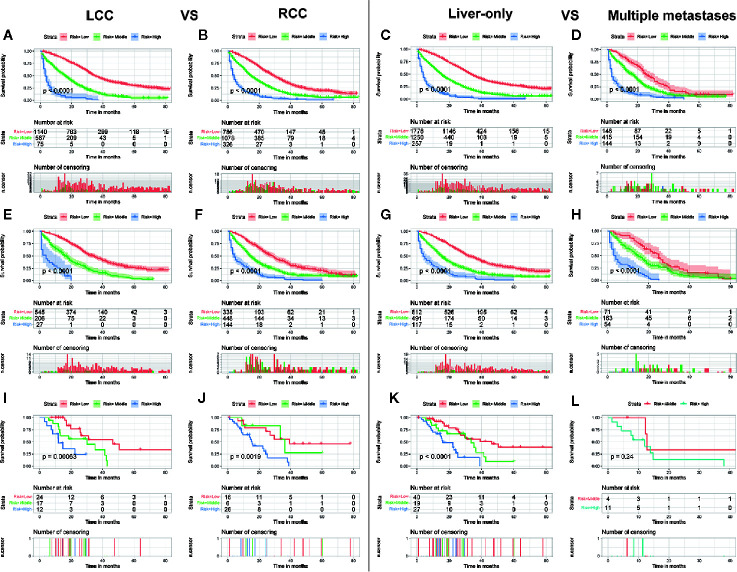
Subgroup analysis of LCC and RCC in the training set **(A, B)**, internal validation set **(E, F)**, and external validation set **(I, J)**; Subgroup analysis of liver-only and multiple metastases in the training set **(C, D)**, internal validation set **(G, H)**, and external validation set **(K, L)**.

**Figure 4:**
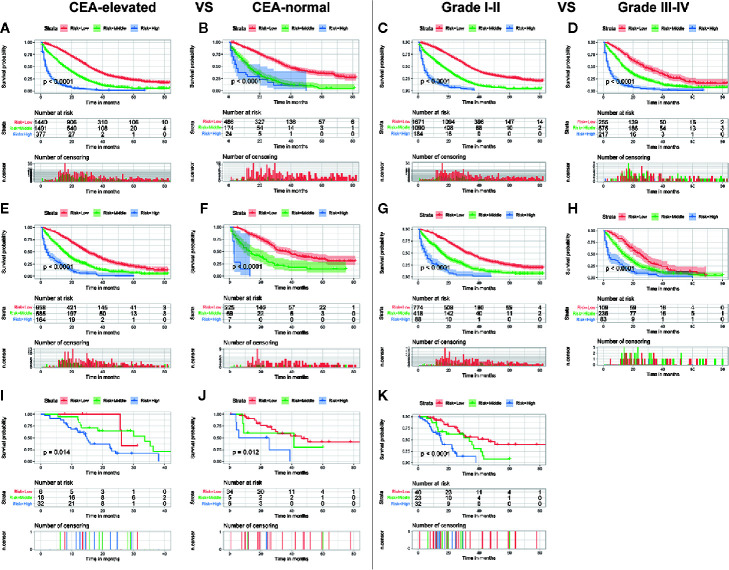
Subgroup analysis of CEA-elevated and CEA-normal in the training set **(A, B)**, internal validation set **(E, F)**, and external validation set **(I, J)**; Subgroup analysis of grade I–II and grade III–IV in the training set **(C, D)**, internal validation set **(G, H)**, and external validation set **(K)**.

Although the ability of the nomogram has been confirmed in both training and validation sets, it remains unclear in subgroups. Hence, to further verify the stability and performance of the nomogram from different dimensions, we divided patients into different subgroups based on tumor site, CEA, the number of distant metastasis sites, and grade. As shown in **Figures 3**, **4**, no matter in training or validation sets, risk stratification can divide patients with different OS into the subgroups of LCC, RCC, CEA-elevated, and CEA-normal, which indicated that the nomogram was effective for the distinction of the prognosis in different CCLM patients subgroups. However, in the multiple metastases subgroup of the external validation set, the survival of patients in the three risk groups was not significantly different (p=0.24), which may be attributed to the relatively small sample size (n=15) (**Figure 3L**). For the grade subgroups, because there are few patients (n=6, all of them belong to the high-risk group) in grade III-IV, we only analyzed the survival status of patients in grade I-II (**Figure 4K**).

### Comparison of Predictive Accuracy

**Figure 5:**
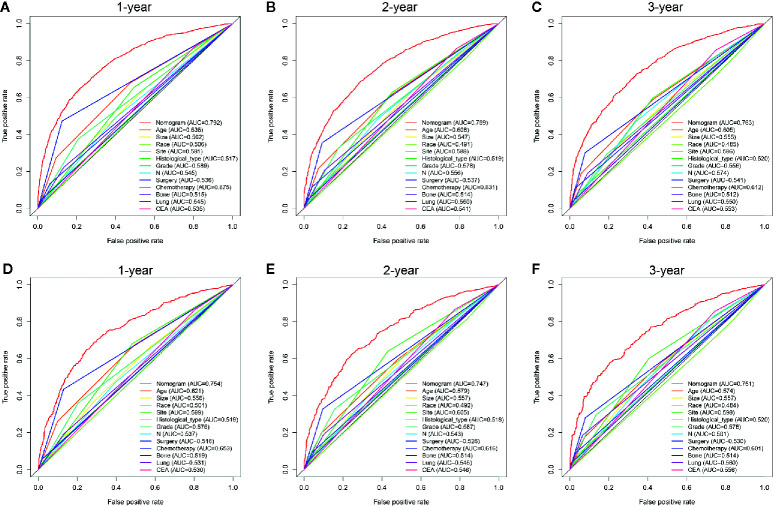
Comparison of predictive accuracy between the nomogram and single independent factors in the training **(A–C)** and internal validation sets **(D–F)**.

AS shown in **Figure 5**, the AUC values of every independent prognostic factor were higher than 0.5, including the training set and the two validation sets. By comparing the predictive power between the nomogram and all independent factors, we found that the AUC value of the nomogram was higher than every single factor in 1-, 2- and 3-years, suggesting the effectiveness of the nomogram.

## Discussion

CC is a highly invasive cancer that is prone to distant metastases, and the most common distant metastatic pattern is liver metastasis. Thus, we included a range of clinicopathological variables to construct a clinical prognostic nomogram for OS of CCLM patients, which achieved considerable discrimination ability and calibration accuracy when applied to the validation cohorts. According to the nomogram risk stratification model, patients in the training or verification group could be effectively divided into three groups (high-, middle- and low-risk groups) with the significant OS. In addition, we included different treatments in the nomogram to clinicians for more facile individual survival prediction.

Although some predictive models have been established in previous studies, we think our study improves upon the previous work. Compared with the study of Wu et al. (20), improvements in ours are as follows. First, from the perspective of the subject, CC and RC patients with liver-only metastasis were included in the study of Wu et al. Although the liver is the most common metastatic site of CC and RC, different molecular developmental mechanisms and metastatic patterns require different staging methods and treatments between CC and RC (21–23). Therefore, our study only included CC to provide a more accurate prediction of prognosis for CCLM. Second, the study of Wu et al. focused on CRC patients with liver-only metastasis, but it was discovered that multiple metastases occur in approximately 20% of CRC patients (24). Thus, this part of patients cannot be predicted through the nomogram established by Wu et al., while the nomogram we constructed can be used. More importantly, subgroup analyses of both liver-only patients and multiple metastases patients showed good performance of our nomogram, which further confirm the improvement of our model. Then, among treatment factors, only surgery was included in the study of Wu et al. Whether it was used as a disease treatment method or as an adjuvant treatment, chemotherapy was considered to be beneficial for CCLM patient’s survival (25, 26). Thus, the factor of chemotherapy was also included in our study and was identified as a protective factor. Finally, we conducted external validation on the established nomogram, which is important and strong evidence.

From the perspective of the patient’s condition, older age, the race of black, lung metastasis, and bone metastasis are independent prognostic factors of CCLM patients’ prognosis. Elderly patients are often accompanied by dysfunction, malnutrition, and comorbidity, which prompts the physicians to choose a less active treatment or shorten the course of treatment and affect the outcome of treatment (27–29). Meanwhile, it was reported that the prognosis of liver metastasis alone was different from multiple metastases in the elderly group, but not in the middle-aged group in a previous study (30). And this study also found that CCLM patients with extrahepatic metastasis had shorter survival times than patients with liver-only metastases, including lung metastasis and bone metastasis (30). The results in our study suggested that the metastatic sites of lung and bone are independently associated with the prognosis of CCLM patients, which was consistent with the conclusion of previous studies (31).

From the perspective of the tumor, tumor site, tumor size, histological type, N stage, histological grade, and CEA level were determined as independent prognostic factors of CCLM. Previous studies reported that RCC had lower OS and disease-free survival than LCC (32, 33), which may be associated with RCC usually presents with a diagnosis of a more advanced stage (34). And another reason may be that microsatellite instability and mutations of KRAS and BRAF are more common in RCC patients (35). Lymph node metastasis is a common form of metastasis in CC, and high rates are also associated with a high risk of multiple metastatic sites and worse differentiation (36). Through the above indirect effects, the prognosis of patients is poor, which proves that the prognosis is related to the N stage. And the conclusion of the higher N stage, the worse the prognosis was consistent with our study (37). However, in the study of Wang et al, only the N1 stage was independently associated with the prognosis of stage IV CRC. While in our study, both N1 and N2 stages were the prognostic factors of CCLM, which may be contributed to the difference between CC and RC and the difference in metastatic patterns. Based on many studies, CEA was also closely related to the survival of advanced CRC patients with liver metastases (38). This conclusion coincided with the results shown in the present study.

From the perspective of treatments, the traditional treatment for patients with stage I–III CC is surgery combined with adjuvant chemotherapy. Partial or total colectomy is performed in 84% of patients with stage I and II CC, while 67% in stage III (39). And adjuvant chemotherapy within 8 weeks after surgery significantly improves the prognosis of patients. Besides, a recent study has found that adjuvant radiotherapy may benefit CC patients, implying that radiotherapy may also be a treatment option for CC patients. With the advancement of treatment, surgery has also become the standard treatment option for CCLM patients, which can improve patients’ outcomes. In clinical practice, partial colectomy and total/subtotal colectomy are more effective for CCLM patients than those without surgery. Additionally, chemotherapy is also an important treatment approach for CCLM patients to significantly prolong the survival time, such as 5-fluorouracil/leucovorin (5-FU/lv), capecitabine, irinotecan, and oxaliplatin (40). As neoadjuvant therapy, chemotherapy can also promote the likelihood of resectability and treat micro-metastases (41, 42). Moreover, as the postoperative adjuvant therapy, the previous study reported that chemotherapy was related to OS and DFS of CRC patients with liver metastasis (43). However, more than 80% of CCLM patients are unresectable, and the prognosis of these patients can also be improved with different chemotherapy regimens (6, 44). Thus, as with our results, surgery and chemotherapy can improve the outcomes of CCLM patients.

In the present study, the nomogram could be used to effectively predict the prognosis of CCLM patients. However, some limitations should be stated. Firstly, this is a retrospective study based on a publicly available database, which made it susceptible to the inherent weaknesses of retrospective data collection. Besides, specific information of liver metastases associated with the prognosis of CCLM, such as the large size, more than three liver metastases, and presence of bi-lobar metastases, is a lack in the SEER database. Secondly, most patients in the external invalidation set were of other races (Asian) and have received chemotherapy, which may produce selection bias. Thirdly, the sample size of the external validation set was not very large, So, other validation cohorts with a larger sample size for the predictive nomogram are indispensable.

## Conclusion

In summary, we found that higher age, the race of black, larger tumor size, higher grade, histological type of mucinous adenocarcinoma and signet ring cell carcinoma, higher N stage, RCC, lung metastasis, bone metastasis, without surgery, without chemotherapy, and elevated CEA were independently associated with poor prognosis of CCLM patients. A nomogram incorporating the above 12 predictors could accurately predict the prognosis of CCLM patients.

## Data Availability Statement

Publicly available datasets were analyzed in this study. This data can be found here: Surveillance, Epidemiology, and End Results (SEER) database (https://seer.cancer.gov/).

## Ethics Statement

Written informed consent was obtained from the individual(s) for the publication of any potentially identifiable images or data included in this article.

## Author Contributions

CL and TW designed the research; CH performed the research and analyzed results; CL and JH edited the manuscript; TW and ZL provided critical comments and revised the manuscript; GZ, JQ, YC, XQ, and YL collected and organized data; GZ added the data, sorted them out and analyzed them in the revised manuscript; KX wrote the revised manuscript; All authors contributed to the article and approved the submitted version.

## Funding

This study was funded by the National Natural Science Foundation of China (No. 31770963); National Science and Technology Major Project of the Ministry of Science and Technology of China (No. 2017ZX09304025); Technological Special Project of Liaoning Province of China (2019020176-JH1/103); Science and Technology Plan Project of Liaoning Province; NO.2013225585).

## Conflict of Interest

The authors declare that the research was conducted in the absence of any commercial or financial relationships that could be construed as a potential conflict of interest.

## Abbreviations

CC, colon cancer; CCLM, colon cancer with liver metastasis; SEER, the Surveillance, Epidemiology and End Results; OS, overall survival; MAC, mucinous adenocarcinoma; SRCC, signet ring cell carcinoma; CEA, carcinoembryonic antigen; ROC, receiver operating characteristic; AUC, area under the curve; DCA, decision curve analysis; LCC, left-side colon cancer; RCC, right-side colon cancer; RC, rectal cancer; CRC, colorectal cancer; DFS, disease-free survival.

## References

[1] Siegel RL Miller KD Jemal A. Cancer statistics, 2019. CA: Cancer J Clin (2019) 69(1):7–34. 10.3322/caac.21551 30620402

[2] Arnold M Sierra MS Laversanne M Soerjomataram I Jemal A Bray F. Global patterns and trends in colorectal cancer incidence and mortality. Gut (2017) 66(4):683–91. 10.1136/gutjnl-2015-310912 26818619

[3] Dekker E Tanis PJ Vleugels JLA Kasi PM Wallace MB. Colorectal cancer. Lancet (London England) (2019) 394(10207):1467–80. 10.1016/s0140-6736(19)32319-0 31631858

[4] Hu H Wu Z Wang C Huang Y Zhang J Cai Y. Duration of FOLFOX Adjuvant Chemotherapy in High-Risk Stage II and Stage III Colon Cancer With Deficient Mismatch Repair. Front Oncol (2020) 10:579478. 10.3389/fonc.2020.579478 33344234 PMC7747753

[5] McLaughlin C Kim NK Bandyopadhyay D Deng X Kaplan B Matin K. Adjuvant radiation therapy for T4 non-rectal colon adenocarcinoma provides a cause-specific survival advantage: A SEER database analysis. Radiother Oncol J Eur Soc Ther Radiol Oncol (2019) 133:50–3. 10.1016/j.radonc.2018.11.026 PMC10105524 30935581

[6] Manfredi S Lepage C Hatem C Coatmeur O Faivre J Bouvier AM. Epidemiology and management of liver metastases from colorectal cancer. Ann Surg (2006) 244(2):254–9. 10.1097/01.sla.0000217629.94941.cf PMC1602156 16858188

[7] Siegel RL Miller KD Fedewa SA Ahnen DJ Meester RGS Barzi A. Colorectal cancer statistics, 2017. CA: Cancer J Clin (2017) 67(3):177–93. 10.3322/caac.21395 28248415

[8] Van Cutsem E Cervantes A Adam R Sobrero A Van Krieken JH Aderka D. ESMO consensus guidelines for the management of patients with metastatic colorectal cancer. Ann Oncol (2016) 27(8):1386–422. 10.1093/annonc/mdw235 27380959

[9] Engstrand J Nilsson H Strömberg C Jonas E Freedman J. Colorectal cancer liver metastases - a population-based study on incidence, management and survival. BMC Cancer (2018) 18(1):78. 10.1186/s12885-017-3925-x 29334918 PMC5769309

[10] Jones RP Kokudo N Folprecht G Mise Y Unno M Malik HZ. Colorectal Liver Metastases: A Critical Review of State of the Art. Liver Cancer (2016) 6(1):66–71. 10.1159/000449348 27995090 PMC5159727

[11] Wang J Li S Liu Y Zhang C Li H Lai B. Metastatic patterns and survival outcomes in patients with stage IV colon cancer: A population-based analysis. Cancer Med (2020) 9(1):361–73. 10.1002/cam4.2673 PMC6943094 31693304

[12] Zheng P Lai C Yang W Guo J Xiao S Chen Z. Nomogram predicting cancer-specific survival in elderly patients with stages I-III colon cancer. Scand J Gastroenterol (2020) 55(2):202–8. 10.1080/00365521.2020.1720280 32008420

[13] Kawai K Nozawa H Hata K Kiyomatsu T Tanaka T Nishikawa T. Nomogram Predicting Survival After Recurrence in Patients With Stage I to III Colon Cancer: A Nationwide Multicenter Study. Dis Colon Rectum (2018) 61(9):1053–62. 10.1097/dcr.0000000000001167 30086054

[14] Kazem MA Khan AU Selvasekar CR. Validation of nomogram for disease free survival for colon cancer in UK population: A prospective cohort study. Int J Surg (London England) (2016) 27:58–65. 10.1016/j.ijsu.2015.12.069 26796369

[15] Zhang Y Ma J Zhang S Deng G Wu X He J. A prognostic analysis of 895 cases of stage III colon cancer in different colon subsites. Int J Colorectal Dis (2015) 30(9):1173–83. 10.1007/s00384-015-2273-z 26054387

[16] Park HS Lloyd S Decker RH Wilson LD Yu JB. Overview of the Surveillance, Epidemiology, and End Results database: evolution, data variables, and quality assurance. Curr Probl Cancer (2012) 36(4):183–90. 10.1016/j.currproblcancer.2012.03.007 22481006

[17] Camp RL Dolled-Filhart M Rimm DL. X-tile: a new bio-informatics tool for biomarker assessment and outcome-based cut-point optimization. Clin Cancer Res (2004) 10(21):7252–9. 10.1158/1078-0432.ccr-04-0713 15534099

[18] Wang Y Li J Xia Y Gong R Wang K Yan Z. Prognostic nomogram for intrahepatic cholangiocarcinoma after partial hepatectomy. J Clin Oncol (2013) 31(9):1188–95. 10.1200/jco.2012.41.5984 23358969

[19] Stojadinovic A Bilchik A Smith D Eberhardt JS Ward EB Nissan A. Clinical decision support and individualized prediction of survival in colon cancer: bayesian belief network model. Ann Surg Oncol (2013) 20(1):161–74. 10.1245/s10434-012-2555-4 22899001

[20] Wu Q Wang WJ Huang YQ Fang SY Guan YJ. Nomograms for estimating survival in patients with liver-only colorectal metastases: A retrospective study. Int J Surg (London England) (2018) 60:1–8. 10.1016/j.ijsu.2018.10.032 30366096

[21] Paschke S Jafarov S Staib L Kreuser ED Maulbecker-Armstrong C Roitman M. Are Colon and Rectal Cancer Two Different Tumor Entities? A Proposal to Abandon the Term Colorectal Cancer. Int J Mol Sci (2018) 19(9):2577. 10.3390/ijms19092577 PMC6165083 30200215

[22] Kalady MF Sanchez JA Manilich E Hammel J Casey G Church JM. Divergent oncogenic changes influence survival differences between colon and rectal adenocarcinomas. Dis Colon Rectum (2009) 52(6):1039–45. 10.1007/DCR.0b013e31819edbd4 19581844

[23] Tamas K Walenkamp AM de Vries EG van Vugt MA Beets-Tan RG van Etten B. Rectal and colon cancer: Not just a different anatomic site. Cancer Treat Rev (2015) 41(8):671–9. 10.1016/j.ctrv.2015.06.007 26145760

[24] Qiu M Hu J Yang D Cosgrove DP Xu R. Pattern of distant metastases in colorectal cancer: a SEER based study. Oncotarget (2015) 6(36):38658–66. 10.18632/oncotarget.6130 PMC4770727 26484417

[25] Leone F Artale S Marino D Cagnazzo C Cascinu S Pinto C. Panitumumab in combination with infusional oxaliplatin and oral capecitabine for conversion therapy in patients with colon cancer and advanced liver metastases. MetaPan Study Cancer (2013) 119(19):3429–35. 10.1002/cncr.28223 23868516

[26] Sereno Moyano M Casado Sáenz E de Castro-Carpeño J Belda-Iniesta C. The combination of FOLFOX4 and bevacizumab may enable salvage surgery of unresectable liver metastases in colon cancer. Anti-Cancer Drugs (2009) 20 Spec No 1:S4–6. 10.1097/01.cad.0000349777.01991.25 19352109

[27] Burdett N Vincent AD O’Callaghan M Kichenadasse G. Competing Risks in Older Patients With Cancer: A Systematic Review of Geriatric Oncology Trials. J Natl Cancer Inst (2018) 110(8):825–30. 10.1093/jnci/djy111 30011032

[28] Lee L Cheung WY Atkinson E Krzyzanowska MK. Impact of comorbidity on chemotherapy use and outcomes in solid tumors: a systematic review. J Clin Oncol (2011) 29(1):106–17. 10.1200/jco.2010.31.3049 21098314

[29] Foster JA Salinas GD Mansell D Williamson JC Casebeer LL. How does older age influence oncologists’ cancer management? Oncol (2010) 15(6):584–92. 10.1634/theoncologist.2009-0198 PMC3227998 20495217

[30] Yang L Yang X He W Liu S Jiang C Xie K. Comparisons of metastatic patterns of colorectal cancer among patients by age group: a population-based study. Aging (Albany NY) (2018) 10(12):4107–19. 10.18632/aging.101700 PMC6326680 30594909

[31] Baek SJ Hur H Min BS Baik SH Lee KY Kim NK. The Characteristics of Bone Metastasis in Patients with Colorectal Cancer: A Long-Term Report from a Single Institution. World J Surg (2016) 40(4):982–6. 10.1007/s00268-015-3296-x 26541868

[32] Signorelli C Chilelli MG Sperduti I Giacinti S Amodio PM Palmieri RM. Correlation of Tumor Location to Clinical Outcomes in Colorectal Cancer: A Single-institution Retrospective Analysis. Anticancer Res (2019) 39(9):4917–24. 10.21873/anticanres.13679 31519596

[33] Lim DR Kuk JK Kim T Shin EJ. Comparison of oncological outcomes of right-sided colon cancer versus left-sided colon cancer after curative resection: Which side is better outcome? Medicine (2017) 96(42):e8241. 10.1097/md.0000000000008241 29049212 PMC5662378

[34] Alexiusdottir KK Möller PH Snaebjornsson P Jonasson L Olafsdottir EJ Björnsson ES. Association of symptoms of colon cancer patients with tumor location and TNM tumor stage. Scand J Gastroenterol (2012) 47(7):795–801. 10.3109/00365521.2012.672589 22506981

[35] Nitsche U Stögbauer F Späth C Haller B Wilhelm D Friess H. Right Sided Colon Cancer as a Distinct Histopathological Subtype with Reduced Prognosis. Dig Surg (2016) 33(2):157–63. 10.1159/000443644 26824772

[36] Derwinger K Gustavsson B. A study of lymph node ratio in stage IV colorectal cancer. World J Surg Oncol (2008) 6:127. 10.1186/1477-7819-6-127 19046414 PMC2633268

[37] Wang X Mao M Xu G Lin F Sun P Baklaushev VP. The incidence, associated factors, and predictive nomogram for early death in stage IV colorectal cancer. Int J Colorectal Dis (2019) 34(7):1189–201. 10.1007/s00384-019-03306-1 31089875

[38] Thomas P Toth CA Saini KS Jessup JM Steele G Jr. The structure, metabolism and function of the carcinoembryonic antigen gene family. Biochim Biophys Acta (1990) 1032(2-3):177–89. 10.1016/0304-419x(90)90003-j 2261493

[39] Miller KD Siegel RL Lin CC Mariotto AB Kramer JL Rowland JH. Cancer treatment and survivorship statistics, 2016. CA: Cancer J Clin (2016) 66(4):271–89. 10.3322/caac.21349 27253694

[40] Brandi G De Lorenzo S Nannini M Curti S Ottone M Dall’Olio FG. Adjuvant chemotherapy for resected colorectal cancer metastases: Literature review and meta-analysis. World J Gastroenterol (2016) 22(2):519–33. 10.3748/wjg.v22.i2.519 PMC4716056 26811604

[41] Nordlinger B Benoist S. Benefits and risks of neoadjuvant therapy for liver metastases. J Clin Oncol (2006) 24(31):4954–5. 10.1200/jco.2006.07.9244 17075112

[42] Folprecht G Gruenberger T Bechstein WO Raab HR Lordick F Hartmann JT. Tumour response and secondary resectability of colorectal liver metastases following neoadjuvant chemotherapy with cetuximab: the CELIM randomised phase 2 trial. Lancet Oncol (2010) 11(1):38–47. 10.1016/s1470-2045(09)70330-4 19942479

[43] Ychou M Hohenberger W Thezenas S Navarro M Maurel J Bokemeyer C. A randomized phase III study comparing adjuvant 5-fluorouracil/folinic acid with FOLFIRI in patients following complete resection of liver metastases from colorectal cancer. Ann Oncol (2009) 20(12):1964–70. 10.1093/annonc/mdp236 19567451

[44] Goldberg RM Sargent DJ Morton RF Fuchs CS Ramanathan RK Williamson SK. A randomized controlled trial of fluorouracil plus leucovorin, irinotecan, and oxaliplatin combinations in patients with previously untreated metastatic colorectal cancer. J Clin Oncol (2004) 22(1):23–30. 10.1200/jco.2004.09.046 14665611

