# Supplementary figures and images for: A Prognostic Nomogram of Colon Cancer With Liver Metastasis: A Study of the US SEER Database and a Chinese Cohort
Source: Front Oncol. 2021 Feb 26;11:591009. doi: 10.3389/fonc.2021.591009 (PMC7962604; doi:10.3389/fonc.2021.591009)

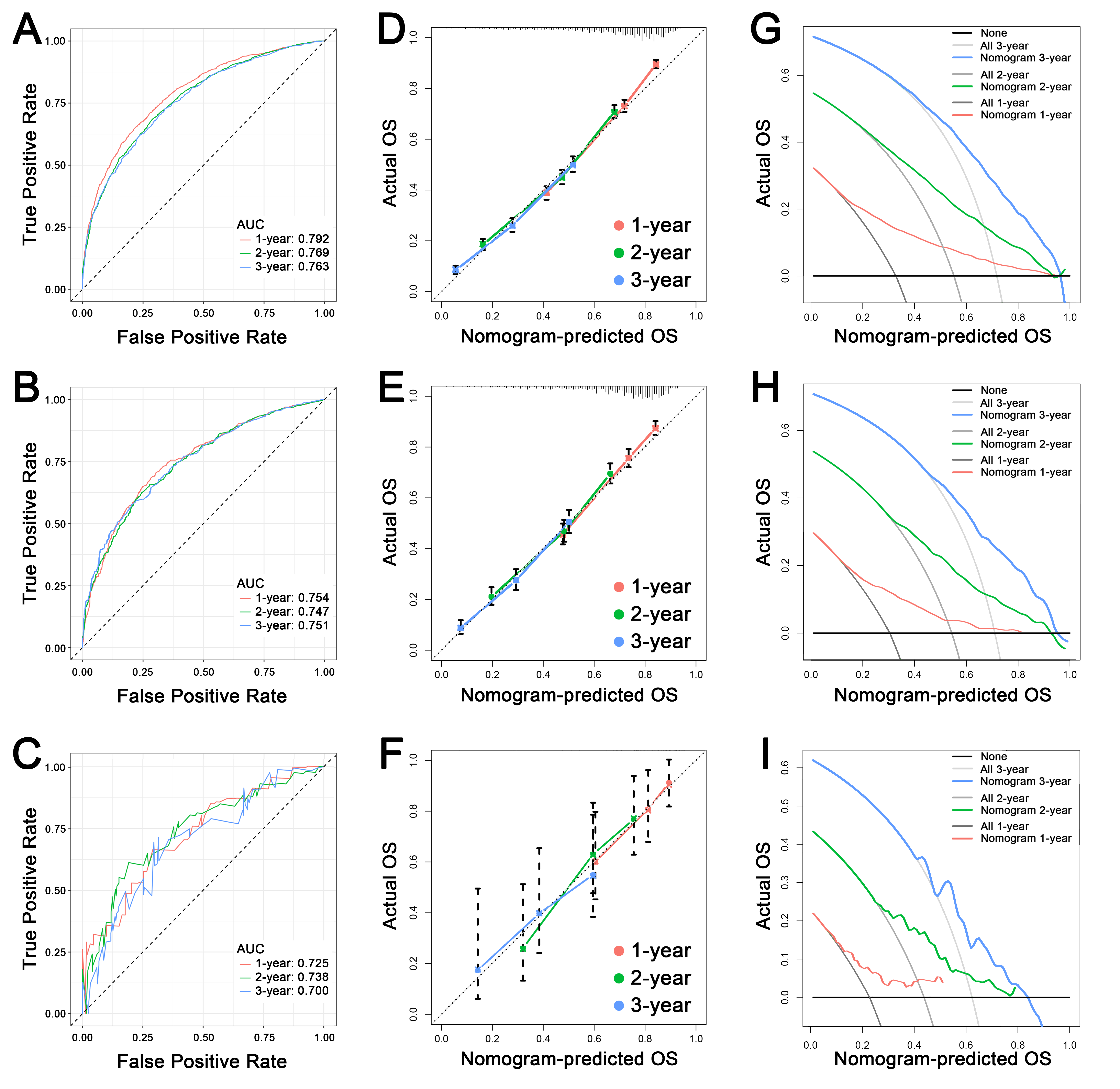

Supplement: Supplementary file 1 [file Image_1.tif]
